# Big data in digital healthcare: lessons learnt and recommendations for general practice

**DOI:** 10.1038/s41437-020-0303-2

**Published:** 2020-03-05

**Authors:** Raag Agrawal, Sudhakaran Prabakaran

**Affiliations:** 1 Department of Genetics, University of Cambridge, Downing Site, Cambridge, CB2 3EH UK; 2 Department of Biology, Columbia University, 116th and Broadway, New York, NY 10027 USA; 3 Department of Biology, Indian Institute of Science Education and Research, Pune, Maharashtra 411008 India; 4 St Edmund’s College, University of Cambridge, Cambridge, CB3 0BN UK

**Keywords:** Genomics, Developing world

## Abstract

Big Data will be an integral part of the next generation of technological developments—allowing us to gain new insights from the vast quantities of data being produced by modern life. There is significant potential for the application of Big Data to healthcare, but there are still some impediments to overcome, such as fragmentation, high costs, and questions around data ownership. Envisioning a future role for Big Data within the digital healthcare context means balancing the benefits of improving patient outcomes with the potential pitfalls of increasing physician burnout due to poor implementation leading to added complexity. Oncology, the field where Big Data collection and utilization got a heard start with programs like TCGA and the Cancer Moon Shot, provides an instructive example as we see different perspectives provided by the United States (US), the United Kingdom (UK) and other nations in the implementation of Big Data in patient care with regards to their centralization and regulatory approach to data. By drawing upon global approaches, we propose recommendations for guidelines and regulations of data use in healthcare centering on the creation of a unique global patient ID that can integrate data from a variety of healthcare providers. In addition, we expand upon the topic by discussing potential pitfalls to Big Data such as the lack of diversity in Big Data research, and the security and transparency risks posed by machine learning algorithms.

## Introduction

The advent of Next Generation Sequencing promises to revolutionize medicine as it has become possible to cheaply and reliably sequence entire genomes, transcriptomes, proteomes, metabolomes, etc. (Shendure and Ji 2008; Topol 2019a). “Genomical” data alone is predicted to be in the range of 2–40 Exabytes by 2025—eclipsing the amount of data acquired by all other technological platforms (Stephens et al. 2015). In 2018, the price for the research-grade sequencing of the human genome had dropped to under $1000 (Wetterstrand 2019). Other “omics” techniques such as Proteomics have also become accessible and cheap, and have added depth to our knowledge of biology (Hasin et al. 2017; Madhavan et al. 2018). Consumer device development has also led to significant advances in clinical data collection, as it becomes possible to continuously collect patient vitals and analyze them in real-time. In addition to the reductions in cost of sequencing strategies, computational power, and storage have become extremely cheap. All these developments have brought enormous advances in disease diagnosis and treatments, they have also introduced new challenges as large-scale information becomes increasingly difficult to store, analyze, and interpret (Adibuzzaman et al. 2018). This problem has given way to a new era of “Big Data” in which scientists across a variety of fields are exploring new ways to understand the large amounts of unstructured and unlinked data generated by modern technologies, and leveraging it to discover new knowledge (Krumholz 2014; Fessele 2018). Successful scientific applications of Big Data have already been demonstrated in Biology, as initiatives such as the Genotype-Expression Project are producing enormous quantities of data to better understand genetic regulation (Aguet et al. 2017). Yet, despite these advances, we see few examples of Big Data being leveraged in healthcare despite the opportunities it presents for creating personalized and effective treatments.

Effective use of Big Data in Healthcare is enabled by the development and deployment of machine learning (ML) approaches. ML approaches are often interchangeably used with artificial intelligence (AI) approaches. ML and AI only now make it possible to unravel the patterns, associations, correlations and causations in complex, unstructured, nonnormalized, and unscaled datasets that the Big Data era brings (Camacho et al. 2018). This allows it to provide actionable analysis on datasets as varied as sequences of images (applicable in Radiology) or narratives (patient records) using Natural Language Processing (Deng et al. 2018; Esteva et al. 2019) and bringing all these datasets together to generate prediction models, such as response of a patient to a treatment regimen. Application of ML tools is also supplemented by the now widespread adoption of Electronic Health Records (EHRs) after the passage of the Affordable Care Act (2010) and Health Information Technology for Economic and Clinical Health Act (2009) in the US, and recent limited adoption in the National Health Service (NHS) (Garber et al. 2014). EHRs allow patient data to become more accessible to both patients and a variety of physicians, but also researchers by allowing for remote electronic access and easy data manipulation. Oncology care specifically is instructive as to how Big Data can make a direct impact on patient care. Integrating EHRs and diagnostic tests such as MRIs, genomic sequencing, and other technologies is the big opportunity for Big Data as it will allow physicians to better understand the genetic causes behind cancers, and therefore design more effective treatment regimens while also improving prevention and screening measures (Raghupathi and Raghupathi 2014; Norgeot et al. 2019). Here, we survey the current challenges in Big Data in healthcare and use oncology as an instructive vignette, highlighting issues of data ownership, sharing, and privacy. Our review builds on findings from the US, UK, and other global healthcare systems to propose a fundamental reorganization of EHRs around unique patient identifiers and ML.

## Current successes of Big Data in healthcare

The UK and the US are both global leaders in healthcare that will play important roles in the adoption of Big Data. We see this global leadership already in oncology (The Cancer Genome Atlas (TCGA), Pan-Cancer Analysis of Whole Genomes (PCAWG)) and neuropsychiatric diseases (PsychENCODE) (Tomczak et al. 2015; Akbarian et al. 2015; Campbell et al. 2020). These Big Data generation and open-access models have resulted in hundreds of applications and scientific publications. The success of these initiatives in convincing the scientific and healthcare communities of the advantages of sharing clinical and molecular data have led to major Big Data generation initiatives in a variety of fields across the world such as the “All of Us” project in the US (Denny et al. 2019). The UK has now established a clear national strategy that has resulted in the likes of the UK Biobank and 100,000 Genomes projects (Topol 2019b). These projects dovetail with a national strategy for the implementation of genomic medicine with the opening of multiple genome-sequencing sites, and the introduction of genome sequencing as a standard part of care for the NHS (Marx 2015). The US has no such national strategy, and while it has started its own large genomic study—“All of Us”—it does not have any plans for implementation in its own healthcare system (Topol 2019b). In this review, we have focussed our discussion on developments in Big Data in Oncology as a method to understand this complex and fast moving field, and to develop general guidelines for healthcare at large.

## Big Data initiatives in the United Kingdom

The UK Biobank is a prospective cohort initiative that is composed of individuals between the ages of 40 and 69 before disease onset (Allen et al. 2012; Elliott et al. 2018). The project has collected rich data on 500,000 individuals, collating together biological samples, physical measures of patient health, and sociological information such as lifestyle and demographics (Allen et al. 2012). In addition to its size, the UK Biobank offers an unparalleled link to outcomes through integration with the NHS. This unified healthcare system allows researchers to link initial baseline measures with disease outcomes, and with multiple sources of medical information from hospital admission to clinical visits. This allows researchers to be better positioned to minimize error in disease classification and diagnosis. The UK Biobank will also be conducting routine follow-up trials to continue to provide information regarding activity and further expanded biological testing to improve disease and risk factor association.

Beyond the UK Biobank, Public Health England launched the 100,000 Genomes project with the intent to understand the genetic origins behind common cancers (Turnbull et al. 2018). The massive effort consists of NHS patients consenting to have their genome sequenced and linked to their health records. Without the significant phenotypic information collected in the UK Biobank—the project holds limited use as a prospective epidemiological study—but as a great tool for researchers interested in identifying disease causing single-nucleotide polymorphisms (SNPs). The size of the dataset itself is its main advance—as it provides the statistical power to discover the associated SNPs even for rare diseases. Furthermore, the 100,000 Genomes Project’s ancillary aim is to stimulate private sector growth in the genomics industry within England.

## Big Data initiatives in the United States and abroad

In the United States, the “All of Us” project is expanding upon the UK Biobank model by creating a direct link between patient genome data and their phenotypes by integrating EHRs, behavioral, and family data into a unique patient profile (Denny et al. 2019). By creating a standardized and linked database for all patients—“All of Us” will allow researchers greater scope than the UK BioBank to understand cancers and discover the associated genetic causes. In addition, “All of Us” succeeds in focusing on minority populations and health, an area of focus that sets it apart and gives it greater clinical significance. The UK should learn from this effort by expanding the UK Biobank project to further include minority populations and integrate it with ancillary patient data such as from wearables—the current UK Biobank has ~500,000 patients that identify as white versus ~12,000 (i.e., just <2.5%) that identified as non-white (Cohn et al. 2017). Meanwhile, individuals of Asian ethnicities made up over 7.5% of the UK population as per the 2011 UK Census, with the proportion of minorities projected to rise in the coming years (O’Brien and Potter-Collins 2015; Cohn et al. 2017).

Sweden too provides an informative example of the power of investment in rich electronic research registries (Webster 2014). The Swedish government has committed over $70 million dollars in funding per annum to expand a variety of cancer registries that would allow researchers insight into risk factors for oncogenesis. In addition, its data sources are particularly valuable for scientists, as each patient’s entries are linked to unique identity numbers that can be cross references with over 90 other registries to give a more complete understanding of a patient’s health and social circumstances. These registries are not limited to disease states and treatments, but also encompass extensive public administrative records that can provide researchers considerable insight into social indicators of health such as income, occupation, and marital status (Connelly et al. 2016). These data sources become even more valuable to Swedish researchers as they have been in place for decades with commendable consistency—increasing the power of long-term analysis (Connelly et al. 2016). Other nations can learn from the Swedish example by paying particular attention to the use of unique patient identifiers that can map onto a number of datasets collected by government and academia—an idea that was first mentioned in the US Health Insurance Portability and Accountability Act of 1996 (HIPAA) but has not yet been implemented (Davis 2019).

China has recently become a leader in implementation and development of new digital technologies, and it has begun to approach healthcare with an emphasis on data standardization and volume. Already, the central government in China has initiated several funding initiatives aimed at pushing Big Data into healthcare use cases, with a particular eye on linking together administrative data, regional claims data from the national health insurance program, and electronic medical records (Zhang et al. 2018). China hopes to do this through leveraging its existing personal identification system that covers all Chinese nationals—similar to the Swedish model of maintaining a variety of regional and national registries linked by personal identification numbers. This is particularly relevant to cancer research as China has established a new cancer registry (National Central Cancer Registry of China) that will take advantage of the nation’s population size to give unique insight into otherwise rare oncogenesis. Major concerns regarding this initiative are data quality and time. China has only relatively recently adopted the International Classification of Diseases (ICD) revision ten coding system, a standardized method for recording disease states alongside prescribed treatments. China is also still implementing standardized record keeping terminologies at the regional level. This creates considerable heterogeneity in data quality—as well as inoperability between regions—a major obstacle in any national registry effort (Zhang et al. 2018). The recency of these efforts also mean that some time is required until researchers will be able to take advantage of longitudinal analysis—vital for oncology research that aims to spot recurrences or track patient survival. In the future we can expect significant findings to come out of China’s efforts to bring hundreds of millions of patient files available to researchers, but significant advances in standards of care and interoperability must be first surpassed.

The large variety of “Big Data” research projects being undertaken around the world are proposing different approaches to the future of patient records. The UK is broadly leveraging the centralization of the NHS to link genomic data with clinical care records, and opening up the disease endpoints to researchers through a patient ID. Sweden and China are also adopting this model—leveraging unique identity numbers issued to citizens to link otherwise disconnected datasets from administrative and healthcare records (Connelly et al. 2016; Cnudde et al. 2016; Zhang et al. 2018). In this way, tests, technologies and methods will be integrated in a way that is specific to the patient but not necessarily to the hospital or clinic. This allows for significant flexibility in the seamless transfer of information between sites and for physicians to take full advantage of all the data generated. The US’ “All of Us” program is similar in integrating a variety of patient records into a single-patient file that is stored in the cloud (Denny et al. 2019). However, it does not significantly link to public administrative data sources, and thus is limited in its usefulness for long-term analysis of the effects of social contributors to cancer progression and risk. This foretells greater problems with the current ecosystem of clinical data—where lack of integration, misguided design, and ambiguous data ownership make research and clinical care more difficult rather than easier.

## Survey of problems in clinical data use

### Fragmentation

Fragmentation is the primary problem that needs to be addressed if EHRs have any hope of being used in any serious clinical capacity. Fragmentation arises when EHRs are unable to communicate effectively between each other—effectively locking patient information into a proprietary system. While there are major players in the US EHR space such as Epic and General Electric, there are also dozens of minor and niche companies that also produce their own products—many of which are not able to communicate effectively or easily with one another (DeMartino and Larsen 2013). The Clinical Oncology Requirements for the EHR and the National Community Cancer Centers Program have both spoken out about the need for interoperability requirements for EHRs and even published guidelines (Miller 2011). In addition, the Certification Commission for Health Information Technology was created to issue guidelines and standards for interoperability of EHRs (Miller 2011). Fast Healthcare Interoperability Resources (FHIR) is the current new standard for data exchange for healthcare published by Health Level 7 (HL7). It builds upon past standards from both HL7 and a variety of other standards such as the Reference Information Model. FHIR offers new principles on which data sharing can take place through RESTful APIs—and projects such as Argonaut are working to expand adoption to EHRs (Chambers et al. 2019). Even with the introduction of the HL7 Ambulatory Oncology EHR Functional Profile, EHRs have not improved and have actually become pain points for clinicians as they struggle to integrate the diagnostics from separate labs or hospitals, and can even leave physicians in the dark about clinical history if the patient has moved providers (Reisman 2017; Blobel 2018). Even in integrated care providers such as Kaiser Permanente there are interoperability issues that make EHRs unpopular among clinicians as they struggle to receive outside test results or the narratives of patients who have recently moved (Leonard and Tozzi 2012).

The UK provides an informative contrast in its NHS, a single government-run enterprise that provides free healthcare at the point of service. Currently, the NHS is able to successfully integrate a variety of health records—a step ahead of the US—but relies on outdated technology with security vulnerabilities such as fax machines (Macaulay 2016). The NHS has recently also begun the process of digitizing its health service, with separate NHS Trusts adopting American EHR solutions, such as the Cambridgeshire NHS trust’s recent agreement with Epic (Honeyman et al. 2016). However, the NHS still lags behind the US in broad use and uptake across all of its services (Wallace 2016). Furthermore, it will need to force the variety of EHRs being adopted to conform to centralized standards and interoperability requirements that allow services as far afield as genome sequencing to be added to a patient record.

### Misguided EHR design

Another issue often identified with the modern incarnation of EHRs is that they are often not helpful for doctors in diagnosis—and have been identified by leading clinicians as a hindrance to patient care (Lenzer 2017; Gawande 2018). A common denominator among the current generation of EHRs is their focus on billing codes, a set of numbers assigned to every task, service, and drug dispensed by a healthcare professional that is used to determine the level of reimbursement the provider will receive. This focus on billing codes is a necessity of the insurance system in the US, which reimburses providers on a service-rendered basis (Essin 2012; Lenzer 2017). Due to the need for every part of the care process to be billed to insurers (of which there are many) and sometimes to multiple insurers simultaneously, EHRs in the US are designed foremost with insurance needs in mind. As a result, EHRs are hampered by government regulations around billing codes, the requirements of insurance companies, and only then are able to consider the needs of providers or researchers (Bang and Baik 2019). And because purchasing decisions for EHRs are not made by physicians, the priority given to patient care outcomes falls behind other needs. The American Medical Association has cited the difficulty of EHRs as a contributing factor in physician burnout and as a waste of valuable time (Lenzer 2017; Gardner et al. 2019). The NHS, due to its reliance on American manufacturers of EHRs, must suffer through the same problems despite its fundamentally different structure.

Related to the problem of EHRs being optimized for billing, not patient care, is their lack of development beyond repositories of patient information into diagnostic aids. A study of modern day EHR use in the clinic notes many pain points for physicians and healthcare teams (Assis-Hassid et al. 2019). Foremost was the variance in EHR use within the clinic—in part because these programs are often not designed with provider workflows in mind (Assis-Hassid et al. 2019). In addition, EHRs were found to distract from interpersonal communication and did not integrate the many different types of data being created by nurses, physician assistants, laboratories, and other providers into usable information for physicians (Assis-Hassid et al. 2019).

### Data ownership

One of the major challenges of current implementations of Big Data are the lack of regulations, incentives, and systems to manage ownership and responsibilities for data. In the clinical space, in the US, this takes the form of compliance with HIPAA, a now decade-old law that aimed to set rules for patient privacy and control for data (Adibuzzaman et al. 2018). As more types of data are generated for patients and uploaded to electronic platforms, HIPAA becomes a major roadblock to data sharing as it creates significant privacy concerns that hamper research. Today, if a researcher is to search for even simple demographic and disease states—they can rapidly identify an otherwise de-identified patient (Adibuzzaman et al. 2018). Concerns around breaking HIPAA prevent complete and open data sharing agreements—blocking a path to the specificity needed for the next generation of research from being achieved, and also throws a wrench into clinical application of these technologies as data sharing becomes bogged down by nebulousness surrounding old regulations on patient privacy. Furthermore, compliance with the General Data Protection Regulation (GDPR) in the EU has hampered international collaborations as compliance with both HIPAA and GDPR is not yet standardized (Rabesandratana 2019).

Data sharing is further complicated by the need to develop new technologies to integrate across a variety of providers. Taking from the example of the Informatics for Integrating Biology and the Bedside (i2b2) program funded by the NIH with Partners Healthcare, it is difficult and enormously expensive to overlay programs on top of existing EHRs (Adibuzzaman et al. 2018). Rather, a new approach needs to be developed to solve the solution of data sharing. Blockchain provides an innovative approach and has been recently explored in the literature as a solution that centers patient control of their data, and also promotes safe and secure data sharing through data transfer transactions secured by encryption (Gordon and Catalini 2018). Companies exploring this mechanism for data sharing include Nebula Genomics, a firm founded by George Church, that is aimed at securing genomic data in blockchain in a way that scales commercially, and can be used for research purposes with permission only from data owners—the patients themselves. Other firms are exploring using a variety of data types stored in blockchain to create predictive models of disease—such as Doc.Ai—but all are centrally based on the idea of a blockchain to secure patient data and ensure private accurate transfer between sites (Agbo et al. 2019). Advantages of blockchain for healthcare data transfer and storage lie in its security and privacy, but the approach has yet to gain widespread use.

## Recommendations for clinical application

### Design a new generation of EHRs

**Fig. 1:**
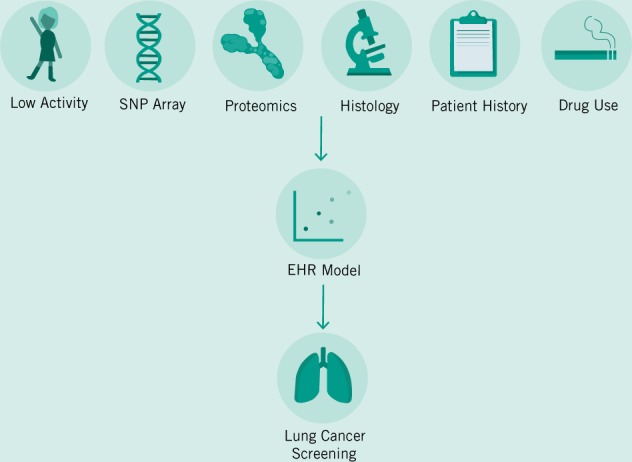
The promise of Big Data is in its ability to prevent disease not just help doctors diagnose them. In this example we demonstrate how many possible factors may come together to better target patients for early screening measures, which can lower aggregate costs for the healthcare system.

It is conceivable that physicians in the near future will be faced with terabytes of data—patients coming to their clinics with years of continuous data monitoring their heart rate, blood sugar, and a variety of other factors (Topol 2019a). Gaining clinical insight from such a large quantity of data is an impossible expectation to place upon physicians. In order to solve this problem of the exploding numbers of tests, assays, and results, EHRs will need to be extended from simply being records of patient–physician interactions and digital folders, to being diagnostic aids (Fig. 1). Companies such as Roche–Flatiron are already moving towards this model by building predictive and analytical tools into their EHRs when they provide them to providers. However, broader adoption across a variety of providers—and the transparency and portability of the models generated will also be vital. AI-based clinical decision-making support will need to be auditable in order to avoid racial bias, and other potential pitfalls (Char et al. 2018). Patients will soon request to have permanent access to the models and predictions being generated by ML models to gain greater clarity into how clinical decisions were made, and to guard against malpractice.

Designing this next generation of EHRs will require collaboration between physicians, patients, providers, and insurers in order to ensure ease of use and efficacy. In terms of specific recommendations for the NHS, the Veterans Administration provides a fruitful approach as it was able to develop its own EHR that compares extremely favorably with the privately produced Epic EHR (Garber et al. 2014). Its solution was open access, public-domain, and won the loyalty of physicians in improving patient care (Garber et al. 2014). However, the VA’s solution was not actively adopted due to lack of support for continuous maintenance and limited support for billing (Garber et al. 2014). While the NHS does not need to consider the insurance industry’s input, it does need to take note that private EHRs were able to gain market prominence in part because they provided a hand to hold for providers, and were far more responsive to personalized concerns raised (Garber et al. 2014). Evidence from Denmark suggests that EHR implementation in the UK would benefit from private competitors implementing solutions at the regional rather than national level in order to balance the need for competition and standardization (Kierkegaard 2013).

### Develop new EHR workflows

**Fig. 2:**
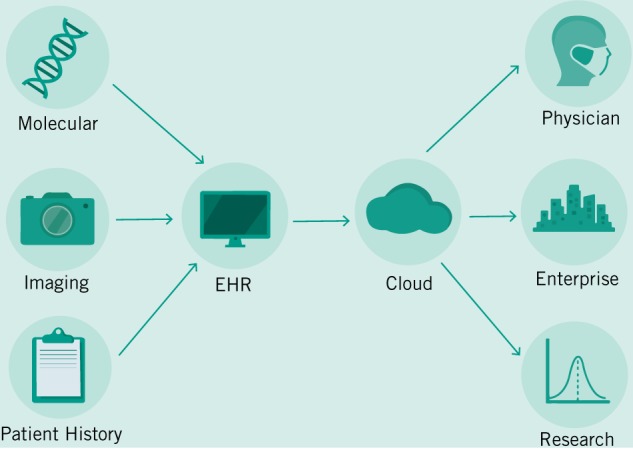
General model of care envisioned. Here, various heterogeneous data types are fed into a centralized EHR system that will be uploaded to a secure digital cloud where it can be de-identified and used by research and enterprise, but primarily by physicians and patients.

Already, researchers and enterprise are developing predictive models that can better diagnose cancers based on imaging data (Bibault et al. 2016). While these products and tools are not yet market ready and are far off from clinical approval—they portend things to come. We envision a future where the job of an Oncologist becomes increasingly interpretive rather than diagnostic. But to get to that future, we will need to train our algorithms much like we train our future doctors—with millions of examples. In order to build this corpus of data, we will need to create a digital infrastructure around Big Data that can both handle the demands of researchers and enterprise as they continuously improve their models—with those of patients and physicians who must continue their important work using existing tools and knowledge. In Fig. 2, we demonstrate a hypothetical workflow based on models provided by other researchers in the field (Bibault et al. 2016; Topol 2019a). This simplified workflow posits EHRs as an integrative tool that can facilitate the capture of a large variety of data sources and can transform them into a standardized format to be stored in a secure cloud storage facility (Osong et al. 2019). Current limitations in HIPAA in the US have prevented innovation in this field, so reform will need to both guarantee the protection of private patient data and the open access to patient histories for the next generation of diagnostic tools. The introduction of accurate predictive models for patient treatment will mean that cancer diagnosis will fundamentally change. We will see the job of oncologists transforming itself as they balance recommendations provided by digital tools that can instantly integrate literature and electronic records from past patients, and their own best clinical judgment.

### Use a global patient ID

While we are already seeing the fruits of decades of research into ML methods, there is a whole new set of techniques that will soon be leaving research labs and being applied to the clinic. This set of “omics”—often used to refer to proteomics, genomics, metabolomics, and others—will reveal even more specificity about a patient’s cancer at lower cost (Cho 2015). However, they like other technologies, will create petabytes of data that will need to be stored and integrated to help physicians.

As the number of tests and healthcare providers diversify—EHRs will need to address the question of extensibility and flexibility. Providers as disparate as counseling offices and MRI imaging centers cannot be expected to use the same software—or even similar software. As specific solutions for diverse providers are created—they will need to interface in a standard format with existing EHRs. The UK Biobank creates a model for these types of interactions in its use of a singular patient ID to link a variety of data types—allowing for extensibility as future iterations and improvements add data sources for the project. Also, Sweden and China are informative examples in their usage of national citizen identification numbers as a method of linking clinical and administrative datasets together (Cnudde et al. 2016; Zhang et al. 2018). Singular patient identification numbers do not yet exist in the US despite their inclusion in HIPAA due to subsequent Congressional action preventing their creation (Davis 2019). Instead private providers have stepped in to bridge the gap, but have also called on the US government to create an official patient ID system (Davis 2019). Not only would a singular patient ID allow for researchers to link US administrative data together with clinical outcomes, but also provide a solution to the questions of data ownership and fragmentation that plague the current system.

## Outlook

Healthcare future will build on the Big Data projects currently being pioneered around the world. The models of data integration being pioneered by the “All of Us” trial and analytics championed by P4 medicine will come to define the patient experience (Flores et al. 2013). However, in this piece we have demonstrated a series of hurdles that the field must overcome to avoid imposing additional burdens on physicians and to deliver significant value. We recommend a set of proposals built upon an examination of the NHS and other publicly administered healthcare models and the US multi-payer system to bridge the gap between the market competition needed to develop these new technologies and effective patient care.

**Fig. 3:**
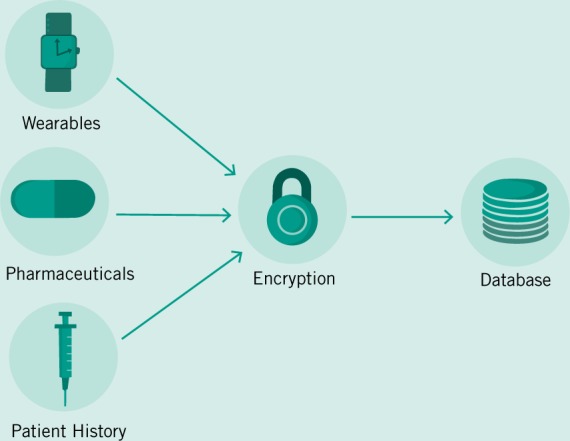
The need for patient data protection is of great concern. Future implementations of Big Data will need to not only integrate data, but also encrypt and de-identify it for secure storage.

**Fig. 4:**
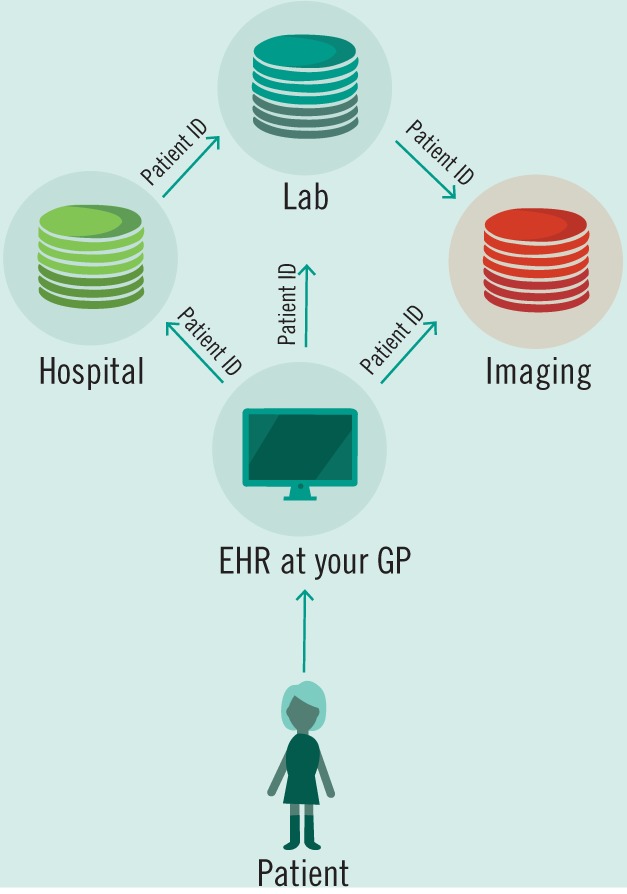
Our recommended healthcare system model. Hypothetical healthcare system design based on unique patient identifiers that function across a variety of systems and providers—linking together disparate datasets into a complete patient profile.

Access to patient data must be a paramount guiding principle as regulators begin to approach the problem of wrangling the many streams of data that are already being generated. Data must both be accessible to physicians and patients, but must also be secured and de-identified for the benefit of research. A pathway taken by the UK Biobank to guarantee data integration and universal access has been through the creation of a single database and protocol for accessing its contents (Allen et al. 2012). It is then feasible to suggest a similar system for the NHS which is already centralized with a single funding source. However, this system will necessarily also be a security concern due to its centralized nature, even if patient data is encrypted (Fig. 3). Another approach is to follow in the footsteps of the US’ HIPAA, which suggested the creation of unique patient IDs over 20 years ago. With a single patient identifier, EHRs would then be allowed to communicate with heterogeneous systems especially designed for labs or imaging centers or counseling services and more (Fig. 4). However, this design presupposes a standardized format and protocol for communication across a variety of databases—similar to the HL7 standards that already exist (Bender and Sartipi 2013). In place of a centralized authority building out a digital infrastructure to house and communicate patient data, mandating protocols and security standards will allow for the development of specialized EHR solutions for an ever diversifying set of healthcare providers and encourage the market needed for continual development and support of these systems. Avoiding data fragmentation as seen already in the US then becomes an exercise in mandating data sharing in law.

**Fig. 5:**
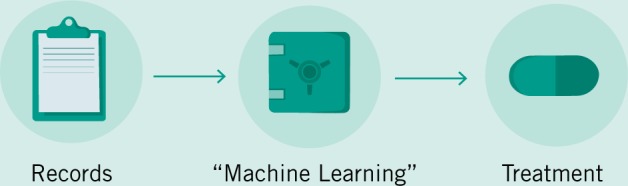
This is an undesirable model for healthcare that can be implemented if regulators are not vigilant. Without transparency in many of the models being implemented as to why and how decisions are being made, there exists room for algorithmic bias and no room for improvement or criticism by physicians. The “black box” of machine learning obscures why decisions are made and what actually affects predictions.

The next problem then becomes the inevitable application of AI to healthcare. Any such tool created will have to stand up to the scrutiny not just of being asked to outclass human diagnoses, but to also reveal its methods. Because of the opacity of ML models, the “black box” effect means that diagnoses cannot be scrutinized or understood by outside observers (Fig. 5). This makes clinical use extremely limited, unless further techniques are developed to deconvolute the decision-making process of these models. Until then, we expect that AI models will only provide support for diagnoses.

**Fig. 6:**
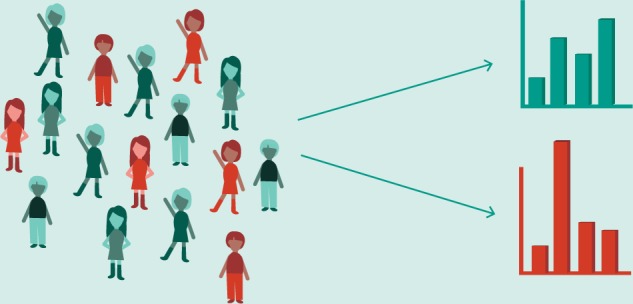
Minority representation in current large-scale experiments integrating across a variety of factors is often lacking. The “All of Us” study will meet this need by specifically aiming to recruit a diverse pool of participants to develop disease models that generalize to every citizen, not just the majority (Denny et al. 2019). Future global Big Data generation projects should learn from this example in order to guarantee equality of care for all patients.

Furthermore, many times AI models simply replicate biases in existing datasets. Cohn et al. 2017 demonstrated clear areas of deficiency in the minority representation of patients in the UK Biobank. Any research conducted on these datasets will necessarily only be able to create models that generalize to the population in them (a largely homogenous white-British group) (Fig. 6). In order to protect against algorithmic bias and the black box of current models hiding their decision-making, regulators must enforce rules that expose the decision-making of future predictive healthcare models to public and physician scrutiny. Similar to the existing FDA regulatory framework for medical devices, algorithms too must be put up to regulatory scrutiny to prevent discrimination, while also ensuring transparency of care.

The future of healthcare will increasingly live on server racks and be built in glass office buildings by teams of programmers. The US must take seriously the benefits of centralized regulations and protocols that have allowed the NHS to be enormously successful in preventing the problem of data fragmentation—while the NHS must approach the possibility of freer markets for healthcare devices and technologies as a necessary condition for entering the next generation of healthcare delivery which will require constant reinvention and improvement to deliver accurate care.

Overall, we are entering a transition in how we think about caring for patients and the role of a physician. Rather than creating a reactive healthcare system that finds cancers once they have advanced to a serious stage—Big Data offers us the opportunity to fine tune screening and prevention protocols to significantly reduce the burden of diseases such as advanced stage cancers and metastasis. This development allows physicians to think more about a patient individually in their treatment plan as they leverage information beyond rough demographic indicators such as genomic sequencing of their tumor. Healthcare is not yet prepared for this shift, so it is the job of governments around the world to pay attention to how each other have implemented Big Data in healthcare to write the regulatory structure of the future. Ensuring competition, data security, and algorithmic transparency will be the hallmarks of how we think about guaranteeing better patient care.
